# Trees by the Roadside

**Published:** 1883-06

**Authors:** 


### TREES BY THE ROADSIDE.

There are miles of road that are dusty and hot in summer which might be greatly improved by the setting of trees upon each side. In narrow streets the trees had better be set inside the wall, but where the streets are wide and the sidewalk wide as well, then the trees may be set between the sidewalk and the street. They should not be set too near together, 40 to 50 feet is near enough when they get large. A good way is to set them every 25 feet, and when they get large enough to touch each other let every second tree be cut out, and that will leave room for the others to grow and spread. The best trees to set in streets are the American elm, sugar, scarlet, silver and Norway maples, American lime or bass. When there is little or no soil a large hole should be dug and loam filled in. Trees should be of at least medium size when set, and should be protected by stakes or otherwise. Set only good trees. Better a few good trees from the nursery, well taken up, that many poor ones from the woods and pasture with poor or mutilated roots. Remember that what is worth doing is worth doing well.

---

He who always exacts the last cent is always a mean man.

Drinking water at meals, impairs digestion by weakening the gastric fluids. Drink water an hour before, or an hour after meals.

Dean Stanley, speaking of the perplexities of life, said: "There are problems of life beyond the power of man to exhaust, and in that certainty of uncertainty it is our privilege to rest. The human mind may and ought to repose as calmly before a confessed and unconquerable difficulty as before a confessed and discovered truth."

Dust.—By Julian Hawthorne. Author of "Bressant," "Sebastian Strome," "Garth," etc. It is noteworthy that this novel of English society in the early part of the present century appears at so nearly the same time with Nathaniel Hawthorne's powerful posthumous novel, "Doctor Grimshawe," dealing with England and America at about the same period. This affords opportunity for interesting comparison, or rather contrast between the weird, inverted, poetic style of the father, and the direct, vigorous, picturesque style of the son. Julian Hawthorne has a marked individuality of his own; and the strong, romantic interest and graphic portraiture of "Dust" will add to his growing fame, as thus far the ablest work from a pen gifted with high qualities, both by heredity and training. With illustrations and portrait of the author. Cloth, decorated, green and silver, $1.25. Published by Fords, Howard & Hulbert, 27 Park Place, New York.

